# Causal relationship between duration of mobile phone use and risk of aneurysmal subarachnoid hemorrhage: A 2-sample Mendelian randomization analysis

**DOI:** 10.1097/MD.0000000000046053

**Published:** 2025-11-21

**Authors:** Wen Gao, Xutang Jiang, Qingxin Lin, Lichao Ye, Xinyue Huang, Yu Xiong, Xiumei Guo, Hanlin Zheng, Chuhan Ke, Weipeng Hu, Feng Zheng

**Affiliations:** a Department of Neurology, The Second Affiliated Hospital, Fujian Medical University, Quanzhou, Fujian, China; b Department of Neurosurgery, The Second Affiliated Hospital, Fujian Medical University, Quanzhou, Fujian, China.

**Keywords:** aneurysmal subarachnoid hemorrhage, causal association, duration of mobile phone use, Mendelian randomization

## Abstract

This study investigates whether the duration of mobile phone use (DMPU) is causally associated with the risk of aneurysmal subarachnoid hemorrhage (aSAH). We pooled data from publicly available genome-wide association studies. DMPU was assessed in European populations (n = 456972), and genome-wide association studies data on patients with aSAH were obtained from the Common Metabolic Disease Knowledge Portal (total n = 337159; cases = 7480; controls = 329679). Inverse-variance weighted was applied as the primary Mendelian randomization (MR) method, and 2-sample MR analyses with sensitivity tests were performed. Twenty-three single nucleotide polymorphisms reaching genome-wide significance were selected as instrumental variables for DMPU. Inverse-variance weighted analysis suggested a causal relationship between excessive DMPU and increased risk of aSAH (odds ratio [OR] = 2.20; 95% confidence interval: 1.26–3.83; *P* = .006). MR-Egger regression indicated that directional pleiotropy was unlikely to bias the results (OR = 12.93; 95% confidence interval:1.15–145.31; *P* = .051). The weighted median method supported the causal relationship between excessive DMPU and increased risk of aSAH (OR = 2.48; 95% Cl: 1.17–5.24; *P* = .018). The Cochrane *Q* test and funnel plot showed no heterogeneity or asymmetry, confirming the robustness of the findings. This study provides evidence supporting a causal relationship between DMPU and aSAH. Excessive mobile phone use may increase the risk of aSAH, with important implications for clinical practice, public health, and policy.

## 1. Introduction

Aneurysmal subarachnoid hemorrhage (aSAH) is a type of stroke caused by the rupture of intracranial aneurysms, with a mortality rate reaching 36%.^[1,2]^ Approximately 500,000 aSAH cases are reported yearly worldwide.^[3]^ Survivors experience critical consequences such as cognitive decline, paralysis, and delayed cerebral ischemia.^[4]^ Therefore, primary prevention is crucial.^[5]^ However, traditional risk factors such as smoking, insomnia, and hypertension partially explain aSAH risks. With the increasing incidence of aSAH,^[6]^ identifying other potential risk factors is essential.^[7]^

The use of mobile phones is widespread and has penetrated into daily life, triggering concerns about their health effects.^[8,9]^ Electromagnetic fields alter the electrical activity of the brain and heart. Mobile phones emit a nonionizing electromagnetic radiation, which is absorbed by body tissues and increases brain glucose metabolism. Therefore, different in vivo studies have shown that systolic, diastolic, and mean arterial blood pressure, as well as total cholesterol, atherosclerosis index, and cardiac Nitric oxide levels, are significantly increased after exposure to electromagnetic field radiation and 2.45-GHz wireless fidelity radiation of dual-transceiver phones.^[10,11]^ Long-term exposure to electromagnetic field radiation is associated with increased arrhythmia and cardiovascular mortality.^[12,13]^ These may be attributed to the increased blood lipid levels, which may induce the elevation of atherosclerosis index, peripheral resistance, or myocardial sympathetic nerve activity. In addition, electromagnetic exposure may affect heart rate and blood pressure through direct and indirect mechanisms. The direct mechanism is related to the influence of the electromagnetic field on the flux and steady state of divalent minerals such as Ca2 + and Zn2+. Exposure to electromagnetic fields accelerates Ca2+/calmodulin-dependent myosin light chain phosphorylation. Through indirect mechanisms, radiofrequency(RF) regulates the autonomic nervous system, plasma catecholamines, and glucocorticoids.^[11,12,14]^

However, the excessive use of mobile phones may cause adverse effects in users, leading to stress, anxiety, insomnia, increased sedentary time, a decline in mental health, and, consequently, cardiovascular diseases and hypertension.^[15–17]^ Previous studies have implicated the use of mobile phones in internal carotid artery dissection, suggesting that prolonged use of mobile phones induces direct mechanical damage to the internal carotid artery.^[18–20]^ In addition, long-term use of mobile phones is associated with a high risk of an increased carotid intima-media thickness, indicating the role of the duration of mobile phone use (DMPU) in cerebrovascular injury.^[21,22]^ Nevertheless, the relationship between DMPU and aSAH remains unclear.^[23]^

To address this gap, we applied Mendelian Randomization (MR), a powerful statistical tool for epidemiological studies. The basic idea of MR is to use genetic variation to assess causal relationships between risk variables and specific diseases. This is based on the premise that individuals inherit genetic variants from their parents at conception randomly and independently of confounders. The principle of randomization determines the objective causal relationship between exposures and outcomes.^[24]^ In this study, we used a 2-sample MR design, leveraging genetic variants associated with DMPU as instrumental variables (IVs) to evaluate its causal relationship with aSAH risk.

## 2. Materials and methods

The present study was designed per the Strengthening the Reporting of Observational Studies in Epidemiology MR guidelines using the checklist provided in the Supplementary Material, Supplemental Digital Content, https://links.lww.com/MD/Q708. As the data from the genome-wide association studies were publicly available, and all primary investigations were granted specific ethical approval and informed consent, supplementary ethical authorization was deemed unnecessary for this study.

### 2.1. Study design

We conducted a 2-sample MR analysis using the aggregated statistics of 2 large-scale genome-wide association studies to investigate the causal effect of DMPU on aSAH risk. We used a 2-sample MR design to randomly assign genetic variations that are associated with mobile phone use as IVs. Different MR methods were applied, and sensitivity analysis was performed to test the validity and robustness of our results.^[25]^ The MR design had 3 key assumptions, namely correlation hypothesis: the genetic variation of IVs had a strong correlation with DMPU; independence hypothesis: single nucleotide polymorphisms (SNPs) were not associated with confounding factors; exclusion restriction hypothesis: SNPs only affected aSAH by excessive DMPU, not through other ways.

### 2.2. Data sources

We searched the publicly available aggregate statistical datasets, including substantial data from GWAS. In the IEU Open GWAS project platform (https://gwas.mrcieu.ac.uk/), we studied the exposure levels of DMPU in Europeans (n = 456,972). The aSAH data (number of cases, 7495; controls, 71,934) in the public metabolic disease knowledge portal database (https://hugeamp.org/downloads.html # CD) was used as the outcome.

### 2.3. Selection and verification of SNPs

Based on 3 criteria, we screened and verified independent SNPs related to DMPU. First, SNPs that are associated with DMPU were selected at a genome-wide significance threshold of *P* <5 × 10^−8^. Subsequently, the independence of the selected SNPs was evaluated based on linkage disequilibrium, excluding SNPs with a high linkage disequilibrium (*r*^2^ >0.001) and those having values close to other SNPs with higher p values. Third, palindromic and incomplete SNPs were removed. Finally, SNPs with *F* statistics >10 were selected, excluding those that were weakly related and reducing the impact of potential bias.

### 2.4. Statistical analysis of 2-sample MR

The 2-sample MR analysis primarily involved the inverse-variance weighting (IVW) method. The weighted median and MR-Egger methods were used to evaluate the causal effect of DMPU on aSAH risk. We used the MR-Egger intercept test to assess horizontal pleiotropy, where a significant intercept (*P* <.05) indicated the presence of pleiotropy. Cochran *Q* statistic was used to assess the heterogeneity between the included IVs, and *P* <.05 indicated significant heterogeneity. In addition, a leave-one-out analysis was performed to investigate if the causal association was primarily influenced by any individual SNP. This analysis involved the systematic removal of each IV and the examination of the resulting impact on the overall causal association. The MR analysis and sensitivity estimation were considered statistically significant at *P* <.05, using a 2-tailed test. All estimates were performed using the “TwoSampleMR version 0.6.6” package in R version 4.4.1.^[26]^

## 3. Results

**Figure 1.**
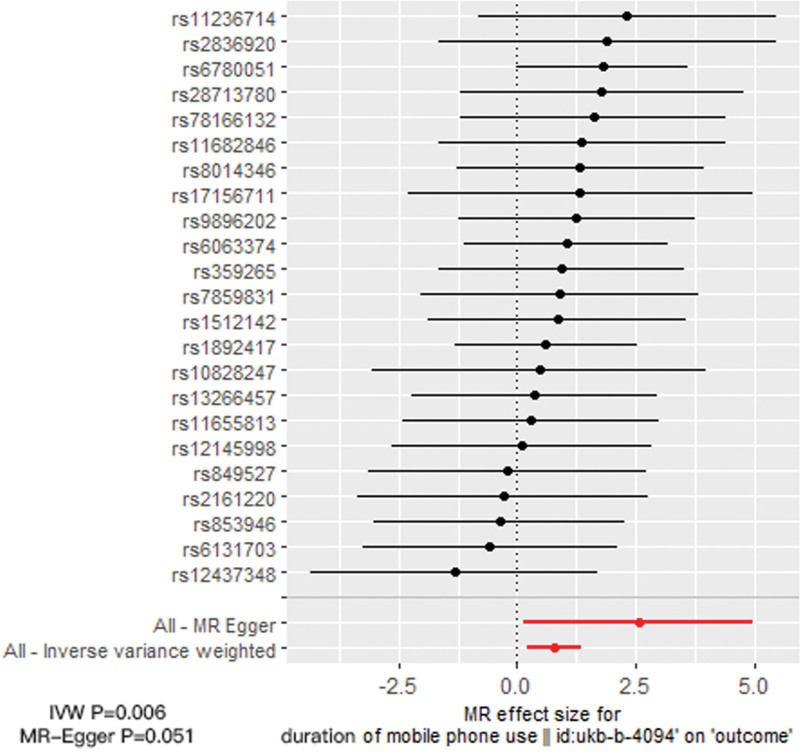
Forest plot of causal influence of SNPs and playing mobile phone for a long time on aSAH. The meaning of the red line is the MR results of MR-Egger test and IVW method. aSAH = aneurysmal subarachnoid hemorrhage, lVW = inverse-variance weighted, MR = Mendelian randomization, SNPs = single nucleotide polymorphisms.

The populations in both databases were primarily Europeans and included males and females. Finally, we identified 23 suitable SNPs with *F*-statistics >10, ruling out weak biases in our results (Table 1). The IVW method revealed a causal relationship between excessive DMPU and an increased aSAH risk (odds ratio [OR] = 2.20; 95% confidence interval [CI]:1.26–3.83; *P* = .006). The weighted median method supported the causal relationship between DMPU and aSAH risk (OR = 2.48; 95% CI: 1.17–5.24; *P* = .018). The MR-Egger analysis indicated no discernible causal relationship between DMPU and aSAH risk (OR = 12.93; 95% CI: 1.15–145.31; *P* = .051). Thus, the association between DMPU and aSAH was inconsistent (Fig. 1). These imply that the IVW and weighted median methods indicated a causal association of excessive DMPU with an increased aSAH risk, while the MR-Egger method failed to substantiate a causal influence. Based on the exceptional precision of the weighted median estimator compared with that of the MR-Egger analysis, the MR analysis suggests a causal association between excessive DMPU and an increased aSAH risk.

**Table 1:** Causal relationship between duration of mobile phone use and the risk of aneurysmal subarachnoid hemorrhage: A 2-sample MR analysis. Summary information for SNPs that were used as genetic instruments for MR analyses of DMPU.

SNP	Effect allele	Other allele	BETA	EAF	SE	*P*	*R* ^2^	*F*
aSAH								
rs10828247	G	A	0.008	0.3179	0.0311	7.39997069733888 × 10^−09^	3.29967430354404 × 10^−05^	15.0790192234278
rs11236714	T	C	−0.0467	0.1603	0.0323	1.79998961724559 × 10^−08^	2.18453904006903 × 10^−05^	19.98290613188523
rs11655813	T	C	0.0051	0.313	0.0248	1.19999655704811 × 10^−09^	3.71199484254763 × 10^−05^	16.9633325100178
rs11682846	T	C	−0.0239	0.3948	0.0271	9.9001095426017 × 10^−10^	4.08329808398409 × 10^−05^	18.6602092063471
rs12145998	T	C	−0.002	0.2355	0.0269	2.90001336905407 × 10^−09^	3.00709885323485 × 10^−05^	13.7419528637343
rs12437348	A	G	−0.0228	0.6184	0.0265	4.20000686246631 × 10^−08^	2.70650497548097 × 10^−05^	12.3682505337715
rs13266457	T	C	−0.0064	0.3309	0.0226	1.70000422215637 × 10^−08^	3.08034741313873 × 10^−05^	14.0766971849977
rs1512142	A	G	−0.0139	0.4289	0.023	8.3000364575513 × 10^−09^	3.58575572234015 × 10^−05^	16.3864155012093
rs17156711	G	A	−0.0242	0.2972	0.0339	4.09996359041765 × 10^−09^	3.16824550606083 × 10^−05^	14.478390199993
rs1892417	C	T	0.0156	0.2841	0.0257	1.2998702373886 × 10^−14^	4.58031897051278 × 10^−05^	20.931642335537
rs2161220	A	G	−0.0062	0.2372	0.0324	4.00000007987242 × 10^−10^	3.19099033959032 × 10^−05^	14.5823338756911
rs2836920	G	T	0.0354	0.3192	0.0339	2.3000114603619 × 10^−10^	4.20830591157495 × 10^−05^	19.2315048446793
rs28713780	C	T	−0.03	0.6189	0.0258	1.09999319893519 × 10^−08^	3.29051044296371 × 10^−05^	15.0371403698854
rs359265	A	G	0.0193	0.6317	0.0277	6.40029512108372 × 10^−13^	5.4001932093222 × 10^−05^	24.6785956004834
rs6063374	G	A	0.0305	0.7518	0.0322	1 × 10^−17^	5.5031627703234 × 10^−05^	25.149186912238
rs6131703	G	A	0.0102	0.3588	0.024	1.89998424621474 × 10^−09^	3.7482722679414 × 10^−05^	17.1291218289351
rs6780051	T	G	0.0712	0.1111	0.0368	6.89922009844049 × 10^−11^	3.01769400016674 × 10^−05^	14.65060360147577
rs78166132	C	T	−0.0491	0.0919	0.0436	4.90004422793237 × 10^−10^	2.4366524147948 × 10^−05^	16.56516485848727
rs7859831	T	C	−0.0209	0.1626	0.0345	2.10000341026661 × 10^−08^	3.64661174589352 × 10^−05^	17.52464559690787
rs8014346	A	G	0.025	0.6156	0.025	3.69998531172859 × 10^−11^	4.76480167420533 × 10^−05^	21.7747517343513
rs849527	G	A	0.0035	0.6175	0.0243	1.49999565138204 × 10^−08^	3.47033495737688 × 10^−05^	15.8589400130643
rs853946	T	C	−0.006	0.4455	0.0217	1.79998961724559 × 10^−08^	3.45520765630278 × 10^−05^	15.7898079976616
rs9896202	C	T	−0.0263	0.3718	0.0265	1.90020300257723 × 10^−13^	5.92298441493414 × 10^−05^	27.0678651063563

The *F* statistic was calculated to estimate sample overlap effects and weak tool bias, where *F* <10 is considered a suspect bias.

aSAH = aneurysmal subarachnoid hemorrhage, DMPU = duration of mobile phone use, MR = Mendelian randomization, SNP = single nucleotide polymorphism.

**Figure 2.**
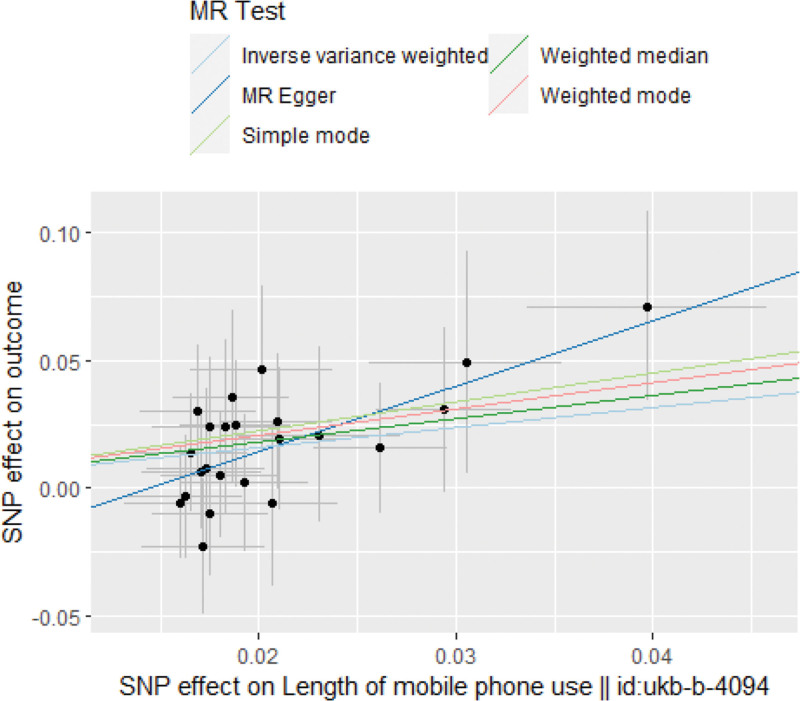
Scatter plot of genetic association and playing mobile phone for a long time.The slope of each line represents the causal relationship of each method. The blue line represents IVW estimation, the green line represents weighted median estimation, and the dark blue line represents MR- Egger estimation. aSAH = aneurysmal subarachnoid hemorrhage, lVW = inverse-variance weighted, MR = Mendel randomization, SNPs = single nucleotide polymorphisms.

**Figure 3.**
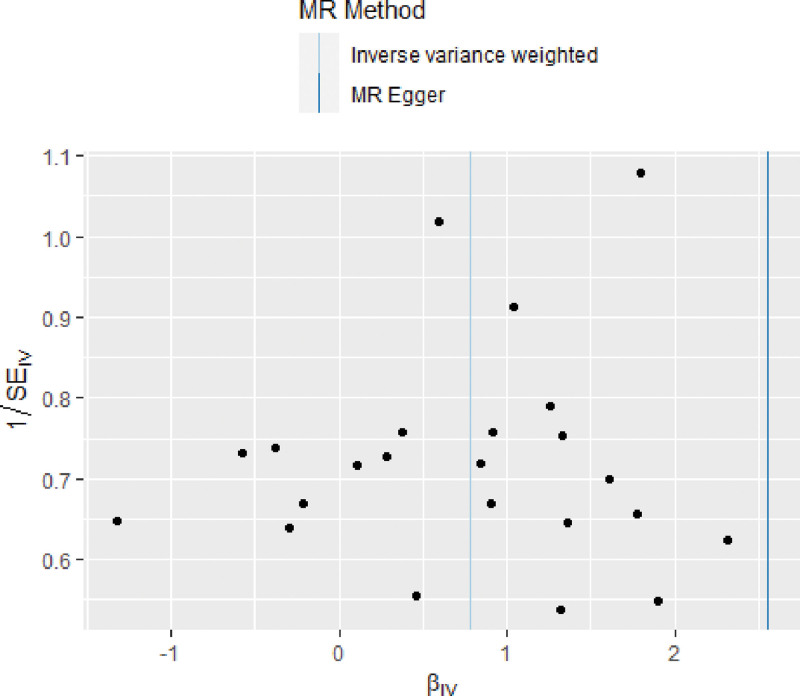
Funnel plot to evaluate heterogeneity. The blue line represents IVW estimation, and the dark blue line represents MR-Egger estimation. lVW = inverse-variance weighted, MR = Mendel randomization.

**Figure 4.**
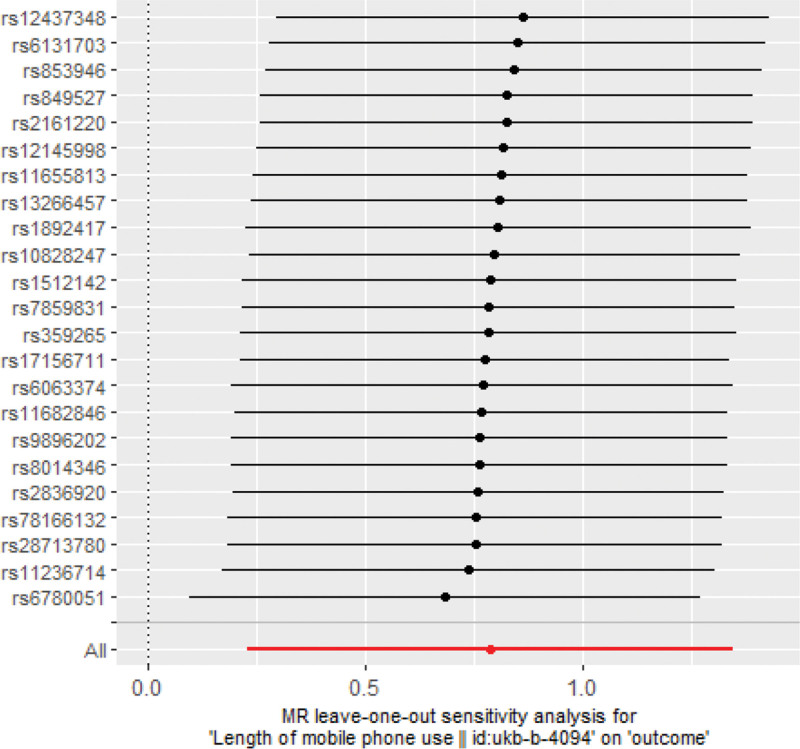
Sensitivity analysis to investigate the possibility of the only SNP-driven causality in aSAH, MR, SNPs. aSAH = aneurysmal subarachnoid hemorrhage, MR = Mendelian randomization, SNPs = single nucleotide polymorphisms.

Heterogeneity and sensitivity assessments using Cochran *Q* test showed no evidence of heterogeneity in IV estimation based on individual variables. Heterogeneity represented the degree of diversity of causal estimates obtained by each SNP, that is, the consistency of causal estimates for all SNPs (Table 2). Therefore, a random-effects IVW method was used to mitigate the impact of heterogeneity. The MR-Egger regression intercept showed no signs of pleiotropy among SNPs (Fig. 2). The scatter and funnel plots did not indicate significant outliers or asymmetry (Fig. 3). In addition, the leave-one-out analysis indicated that there was no single SNP-driven IVW point estimation (Fig. 4). The MR analysis supported a potential causal relationship between excessive DMPU and an increased aSAH risk.

**Table 2:** Mendelian randomization estimates for each method of assessing the causal effect of mobile phone usage time on the risk of aneurysmal subarachnoid hemorrhage.

MR method	No. SNPs	OR	95% CI	Association *P*-value	Cochran *Q* statistic	Cochran *Q P*-value	MR-Egger intercept
Inverse-variance weighted	23	2.20	1.26–3.83	.006	22	.994	0.155
MR-Egger	23	12.93	1.15–145.31	.051	21	.999	NA
Weighted median	23	2.48	1.17–5.24	.018	NA	NA	NA

aSAH = aneurysmal subarachnoid hemorrhage, CI = confidence interval, MR = Mendelian randomization, NA = not available, OR = odds ratio, SNPs = single nucleotide polymorphisms.

## 4. Discussion

In this study, we explored the causal relationship between DMPU and aSAH risk by analyzing the aggregated statistical data of 2 large-scale GWAS datasets with the 2-sample MR method. Our study demonstrated a causal relationship between excessive DMPU and an increased aSAH risk. Our results suggest that reducing the excessive DMPU would benefit aSAH prevention. These findings may influence public health strategies targeting aSAH risks.

Mobile phone usage refers to the duration for which individuals are active on their mobile phones. Recent studies have shown that the average daily mobile phone usage has increased from 2.25 to 4.8 hours.^[27]^ The sharp increase in mobile phone usage has triggered concerns about its potential adverse effects on human health.^[28]^ Excessive DMPU may increase the risk of cardiovascular diseases.^[29]^ Electromagnetic radiation enhances oxidative stress, suggesting a biological pathway for the development of cardiovascular disease.^[14,30]^ However, the relationship between DMPU and aSAH risk is partially elucidated.^[29]^ Therefore, in this study, we used MR analysis to explore the causal relationship between DMPU and aSAH risk. The DMPU refers to the time for which people are active on mobile phones. We believe that when the mobile phone is turned on, regardless of using it to call, access the internet, or carry it on, it induces electromagnetic exposure. Long-term electromagnetic exposure may affect human health. To our knowledge, this is the first study involving a 2-sample MR method to examine the causal relationship between DMPU and aSAH risk.

Our results suggest that excessive DMPU is causally associated with an increased aSAH risk. This could be owing to the effect of mobile phone use on sleep quality,^[31]^ as lack of sleep and an increased aSAH risk are likely correlated.^[7]^ Previous studies have shown that the young population who excessively play games on smartphones are more likely to be overweight or obese.^[32]^ This is caused by an unhealthy diet and sedentary behavior related to mobile phone use. In a meta-analysis of case-control studies, high body mass index was correlated with aSAH.^[7]^ Similarly, poor mental health disrupts cardiovascular metabolic parameters, such as blood pressure, blood lipids, and blood glucose levels, causing autonomic dysfunction and enhancing immune and inflammatory responses, ultimately increasing aSAH risk.^[21]^

Our study has several strengths. First, to our knowledge, this is the first study on the causal relationship between excessive DMPU and aSAH risk at the genetic level. In addition, we used large-scale GWAS data from 2 independent sources as exposures and outcomes, increasing statistical power and reducing the possibility of bias owing to population stratification or confounding factors. Moreover, the 2 datasets comprise Europeans, enabling the reduction of heterogeneity. Furthermore, we used 3 MR methods and sensitivity analyses to estimate and verify the causal effect, confirming the robustness and reliability of the present findings. Finally, we used genetic variation as a long-term stable IV to ensure that DMPU precedes aSAH, eliminating the influence of reverse causality.

However, our study has some limitations. First, GWAS data were used from populations of European ancestry, which may affect the external validity and generalizability. Future studies are required to investigate the causal relationship between excessive DMPU and aSAH risk in different races. Second, the MR approach in this study was based on several assumptions that may not hold. For example, the genetic variation used as an indicator of DMPU may not be independent of confounders of DMPU-aSAH association or may affect aSAH risk through pathways other than excessive DMPU. Although sensitivity analyses were conducted to test the robustness and accuracy of the findings, these methods have limitations. Therefore, our results may have been biased or confounded owing to unknown or unmeasured factors. Third, we used DMPU as a proxy indicator of mobile phone use, which did not capture the content of mobile phone use that may influence aSAH risk. This should be focused on in future studies.

## 5. Conclusion

Our study suggests that excessive DMPU is associated with an increased aSAH risk, which could aid clinical and public health practice and policy. Further longitudinal and experimental studies are required to validate our findings.

**Key Points**

• This study uses MR methods to examine a potential causal association

between the DMPU and aneurysmal subarachnoid hemorrhage risk using genome-wide association study datasets.

• Our study demonstrates that excessive DMPU may be causally associated with an increased aneurysmal subarachnoid hemorrhage risk. We believe that our study makes a significant contribution to the literature because we are the first to use 2-sample MR methods to reduce the bias associated with observational studies and to identify causal relationships.

• We believe that this paper will be of interest to the readership of your journal because our data provides the evidence required to develop clinical and public health policies and provides rationale for longitudinal and experimental studies investigating the mechanisms behind the causality.

## Acknowledgments

The authors would like to thank all participants and researchers for contributing and sharing the GWAS summary data.

## Author contributions

**Conceptualization:** Qingxin Lin.

**Data curation:** Wen Gao, Xutang Jiang, Qingxin Lin.

**Methodology:** Lichao Ye, Xinyue Huang, Yu Xiong, Xiumei Guo, Hanlin Zheng, Chuhan Ke, Weipeng Hu.

**Visualization:** Wen Gao, Xutang Jiang, Qingxin Lin.

**Writing – original draft:** Wen Gao, Xutang Jiang, Qingxin Lin.

**Writing – review & editing:** Lichao Ye, Xinyue Huang, Feng Zheng.

## Supplementary Material


